# Suicide rates and voting choice in the UK's 2016 national Brexit referendum on European Union membership: cross-sectional ecological investigation across England's local authority populations

**DOI:** 10.1192/bjo.2020.32

**Published:** 2020-06-01

**Authors:** Sarah Steeg, Roger T. Webb, Saied Ibrahim, Louis Appleby, Nav Kapur

**Affiliations:** Centre for Mental Health and Safety, Manchester Academic Health Science Centre, University of Manchester, UK; Centre for Mental Health and Safety, Manchester Academic Health Science Centre, University of Manchester; and NIHR Greater Manchester Patient Safety Translational Research Centre, University of Manchester, UK; Centre for Mental Health and Safety, Manchester Academic Health Science Centre, University of Manchester, UK; Centre for Mental Health and Safety, Manchester Academic Health Science Centre, University of Manchester, UK; Centre for Mental Health and Safety, Manchester Academic Health Science Centre, University of Manchester; NIHR Greater Manchester Patient Safety Translational Research Centre, University of Manchester; and Greater Manchester Mental Health NHS Foundation Trust, UK

**Keywords:** Epidemiology, mortality, suicide

## Abstract

**Background:**

Individual- and area-level risk factors for suicide are relatively well-understood but the role of macro social factors such as alienation, social fragmentation or ‘anomie’ is relatively underresearched. Voting choice in the 2016 referendum on the UK's membership of the European Union (EU) provides a potential measure of anomie.

**Aims:**

To examine associations between percentage ‘Leave’ votes in the EU referendum and suicide rates in 2015–2017, the period just prior to, and following, the referendum.

**Method:**

National cross-sectional ecological study of 315 English local authority populations. Associations between voting choice in the EU referendum and age-standardised suicide rates, averaged for the years 2015, 2016 and 2017, were examined.

**Results:**

Overall there was a weak, but statistically significant, positive correlation between the local authority-level percentage ‘Leave’ vote in 2016 and the suicide rate 2015–2017: Pearson's correlation coefficient, *r* = 0.17; *P* = 0.003. This relationship was explained by populations having an older age distribution, being more deprived and lacking ethnic diversity. However, there was divergence (likelihood ratio test for interaction, χ^2^ = 7.2, *P* = 0.007) in the observed associations between London and the provincial regions with Greater London having a moderately strong negative association (*r* = −0.40; *P* = 0.02) and the rest of England a weak positive association (*r* = 0.17; *P* = 0.004).

**Conclusions:**

Deprivation, older age distribution and a lack of ethnic diversity seems to explain raised suicide risk in Brexit-voting communities. A greater sense of alienation among people feeling ‘left behind’/‘left out’ may have had some influence too, although multilevel modelling of individual- versus area-level data are needed to examine these complex relationships. The incongruent ecological relationship observed for London likely reflect its distinct social, economic and health context.

## Background

Suicide is a complex public health problem that requires prevention strategies focused on multiple domains. Risk factors at the individual level are relatively well understood. Being male, experiencing psychiatric disorder, having a history of self-harm, older age and misuse of drugs and alcohol are some characteristics that infer greater risks of suicide.^1^ Broader societal factors are also important. Deprivation is known to be linked with suicide risk, with rates generally higher among people experiencing higher levels of socioeconomic hardship.^2,3^ Periods of economic recession or instability also have adverse effects on suicide rates. For instance, following the 2008–2009 recession in the UK, suicides rose significantly among middle-aged men.^4^ Area-based associations with suicide risk are well characterised,^5^ but the role of macro social factors such as alienation, social fragmentation or ‘anomie’ is relatively underresearched. It has been shown that during economic recession rising unemployment rates and job insecurity can contribute to the weakening of social bonds.^6^ Residents of areas with higher levels of deprivation are more likely to experience cumulative disadvantage, increasing risk of suicide.^7^ However, the concept of anomie – the breakdown of the relationship between an individual and society because of a conflict of values – is not easily measured at a population level nor is it well defined. The degree to which an area can be characterised as being social fragmented has been measured using Congdon's ‘anomie score’, which is essentially a compositional measure of census-based variables indicating a higher likelihood of social incohesion, isolation and instability.^8^ The most socially fragmented areas during the 1980s and 1990s were found to also have the highest suicide rates, even after accounting for deprivation.^9,10^ Recent findings from the Netherlands suggest associations may be more complex, with differences observed in potential influences of social fragmentation by gender and according to individual-level characteristics.^11^

## European Union (EU) membership referendum

Previously, area-level rates of abstention from voting in the general election have been used as a measure of social fragmentation.^9^ However, recent political events in the UK provide another potential measure of anomie. On 23 June 2016 a national referendum was held across the UK, with the following question put to all citizens aged 18 years and over who were eligible to vote: ‘Should the United Kingdom remain a member of the European Union or leave the European Union?’ The referendum delivered an unexpected and momentous result, with 51.9% of the 46.5 million people who voted indicating a preference that the UK ‘Leave’ the EU.^12^ The UK's ‘Brexit’ epoch has been characterised by rancour and acrimony among politicians, the popular media and the general public.^13,14^

Koltai *et al*^15^ recently examined changes in suicide and drug-related deaths according to levels of voting for Brexit at local authority level. Increases in these so-called ‘deaths of despair’ were found to correlate with the per cent of the population that voted in favour of leaving the EU. These authors expounded a conceptual framework linking support for populist political agendas to worsening population health, whereby each share similar antecedents. This ecological association has been demonstrated in the USA, with declining life expectancy linked with level of voting for Donald Trump in the 2016 presidential election.^16^

Goodwin and Heath found that areas characterised by populations of older age, lower educational attainment, greater economic disadvantage and a higher proportion of residents of White British origin were associated with higher proportions of ‘Leave’ voters – collectively, citizens who were perhaps more likely to feel ‘left behind’.^17^ From a spatial perspective, towns and cities that had thrived in recent times were more likely to return a higher ‘Remain’ vote whereas places that had experienced relative economic decline tended to have a higher ‘Leave’ vote.^14^ The voting preference of most Londoners differed markedly from that of most citizens living in the rest of the country, with almost 60% of the voting electorate in the 33 boroughs of the capital city indicating a preference to ‘Remain’.^18^ Greater London has a uniquely diverse demography^19,20^ and a distinct economic context.^21^ Furthermore, suicide rates have been considerably lower there than elsewhere in England.

## Aims and objectives

By considering voting ‘Leave’ as a potential marker of anomie at population level, we aimed to examine the association between voting in favour of Brexit and suicide risk in the 3-year period surrounding the referendum (2015–2017). Our hypothesis was that suicide rates would be higher in local authority populations with a higher percentage ‘Leave’ vote. Our specific objectives were to:

- examine associations between percentage ‘Leave’ votes in the EU referendum suicide rates and for local authority populations;
- explore if any observed association was explained by area-level factors including older age distribution, higher deprivation level and lower ethnic diversity to find potential evidence for anomie;
- compare associations observed across local authority populations in Greater London versus those across the rest of England.

## Method

### Description of the study's data sources

All sources of data that were utilised to conduct this nationwide ecological investigation were reported at local authority level, and all were publicly available free of charge. A spreadsheet containing results from the 2016 EU referendum was downloaded from the UK's Electoral Commission website,^22^ with the same information also being available via BBC News online.^23^

Age-standardised suicide rates per 100 000 people averaged across the three calendar years 2015, 2016 and 2017, and three area-level covariates – Index of Multiple Deprivation (IMD) score, percentage of the population aged 65 years and older and percentage of the population that was of White British ethnicity – were extracted in spreadsheet format from the online Public Health England Local Authority Health Profiles.^24^

The published suicide rates pooled across years 2015–2017 provided an ideal dependent variable for this study because the national EU referendum was held in the middle of this time period, during June 2016. Our goal was to investigate cross-sectional associations rather than to discern putative causal effects of the referendum, its results and its aftermath. Furthermore, suicide rates at local authority level can fluctuate considerably year-on-year because of the rarity of these events, and so aggregation of the estimates over this 3-year period ensured a greater degree of statistical stability and precision.

This study did not require ethical approval as it used only publicly available data reported at local authority level.

### Statistical analyses

Analyses were performed using Stata statistical software version 15.^25^ All but 6 of the 321 local authority areas in England were examined. These six authorities were excluded either because they had low suicide counts during the years 2015–2017 that were too sparse to yield robust statistical analyses, or because of recent local authority mergers. Median age-standardised suicide rates were estimated and reported according to quartiles of the percentage of the total eligible local authority population that voted in favour of exiting the EU.

Pearson's correlation coefficients (*r*) were calculated to indicate the direction and strength of association between the percentage ‘Leave’ vote and the suicide rate, and partial coefficients were also generated in a multivariable model with adjustment for IMD score, percentage of the population aged 65 years and older, and percentage of the population that was White British (factors that may influence suicide rate and voting choice). To examine the associations that were specific to the local authority populations within Greater London versus those in the rest of England, we initially fitted a binary interaction indicator in the full national model, and we then evaluated evidence of an interaction between Greater London and the rest of the country via a likelihood ratio test.

## Results

### Ecological correlations observed across all English local authority populations

The results presented in Table 1 indicate a stepwise interquintile increase in the median age-standardised suicide rates per 100 000 people, 2015–2017 from lowest to highest percentages of local authority area populations that voted in favour of leaving the EU in the 2016 national referendum; i.e. lowest quintile for percentage ‘Leave’ vote (21 to 50%): median rate, 8.98; highest quintile for percentage ‘Leave’ vote (61 to 77%): median rate, 10.0. This weak positive correlation was plotted in relation to the 315 local authority areas in England that were examined – an analysis that confirms the statistical significance of the observed association: *r* = 0.17, *P* = 0.003.

**Table 1:** Median age-standardised suicide rates 2015–2017 per 100 000 people by percentage ‘Leave’ vote quartile in the European Union referendum across all English local authority populations

% ‘Leave’ vote	Range in % ‘Leave’ values per quartile	Median suicide rate	Interquartile range
1st quartile	21–50	8.98	2.34
2nd quartile	50–55	9.03	2.97
3rd quartile	55–61	9.96	3.36
4th quartile	61–77	10.00	2.93

Multivariable adjustments indicate that the correlation was explained by IMD score, percentage of the population aged 65 years and older, and percentage of the population that was White British; indeed, the direction of the observed association was reversed in the adjusted model (*r* = −0.12, *P* = 0.05). The partial correlations (adjusted estimates) for each of the three ecological covariates were similar: IMD score, *r* = 0.11, *P* = 0.04; percentage of the population aged 65 years and older, *r* = 0.13, *P* = 0.02; percentage of the population that was not White British, *r* = 0.10, *P* = 0.08.

### Comparison of the observed ecological relationships between the Greater London local authority populations and those in the rest of England

**Fig. 1:**
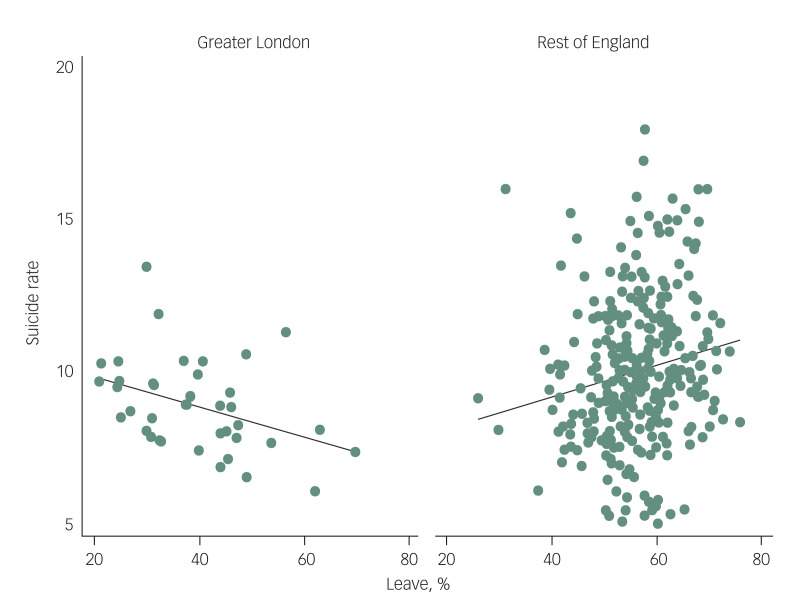
Percentage ‘Leave’ vote in the European Union referendum versus age-standardised suicide rates 2015–2017 per 100 000 people for local authority populations in Greater London versus the rest of England.

Figure 1 shows the comparison in the examined ecological relationships between the local authority populations in Greater London (*n* = 32) against those in the rest of England (*n* = 283). The first plot, the one in the left-hand panel of the figure, indicates a moderately strong negative association in Greater London (*r* = −0.40; *P* = 0.02) whereas, conversely, the other plot in the right-hand panel indicates a weak positive relationship across the rest of England (*r* = 0.17; *P* = 0.004).

We Compared *n* = 32 local authority populations in Greater London with *n* = 283 local authorities in the rest of England. Pearson's correlation coefficients: Greater London *r* = −0.40 (*P* = 0.02); rest of England *r* = 0.17 (*P* = 0.004).

The likelihood ratio test for interaction showed that this marked difference in the ecological associations observed was statistically significant (χ^2^ = 7.2, d.f. = 1, *P* = 0.007). In Greater London, multivariable adjustments for age distribution, deprivation level, percentage of the population that was White British attenuated the negative correlation somewhat to the point that it was no longer statistically significant (Table 2). For the rest of the country, adjustment for all three covariates fully explained the observed positive association, and in fact the direction of association was reversed in this multivariable model.

**Table 2:** Percentage ‘Leave’ vote in the European Union referendum versus age-standardised suicide rates 2015–2017 per 100 000 people for local authority populations in Greater London versus the rest of England – multivariable adjustments

Unadjusted and adjusted models	Local authorities (*n*)	Coefficient	*P*
Greater London			
Crude analysis	32	−0.40	0.02
Multivariate model	32	−0.27	0.16
Rest of England			
Crude analysis – full data-set	283	0.17	0.004
Crude analysis – restricted data-set^a^	254	0.18	0.004
Multivariable model^b^	254	−0.12	0.06

a. Twenty-nine local authorities with suppressed population percentages were excluded from this analysis because of small event counts.

b. Multivariable adjustment for percentage aged 65 years and older, Index of Multiple Deprivation score and percentage of the population that were White British.

## Discussion

As we hypothesised, analysis across all English local authority populations indicated a weak, but statistically significant, positive ecological correlation between the percentage ‘Leave’ vote in the 2016 national EU membership referendum and the age-standardised suicide rate for the years 2015–2017. These findings are consistent with Durkheim's concept of ‘anomic suicide’. This association was explained by having an older age distribution, a greater level of deprivation and a lack of ethnic diversity in the population – three area-level factors that were known to be key drivers of a higher percentage ‘Leave’ vote. However, these factors might themselves be determinents or components of anomie. The study's most striking finding was the marked contrast between the ecological associations observed for the local authority populations of Greater London versus those in the rest of the country – a divergence that is highly unlikely to have arisen by chance. A moderately strong negative association with the suicide rate was observed for the Greater London local authority populations.

### Interpretation of our findings

These findings are concerning, intriguing and topical, but they are also challenging to interpret. However, we believe that the following two inferences can be made.

- The findings point toward a plausible influence of anomie – specifically a greater sense of alienation among ‘left behind’/‘left out’ communities – in explaining why a majority of the populations of the provincial English regions voted in favour of leaving the EU in the 2016 referendum.
- The factors that influence an area's suicide rate may be distinct in Greater London versus the determinants of risk in localities across the rest of England.

The positive correlation found between suicide rates and the percentage ‘Leave’ vote in the 2016 referendum across all English local authority populations seemingly confirms Durkheim's concept of ‘anomic suicide’, likely representing communities where many people feel ‘left behind’ or ‘left out’.

This novel finding is of considerable societal concern because the UK's ‘Brexit’ era could last for many years. The observed associations were explained by existing area-based measures of deprivation, which may themselves be associated with alienation. Strikingly divergent ecological associations were observed between Greater London and the rest of the country as, contrary to the pattern found in the English provincial regions, local authority populations in the capital city with low percentage ‘Leave’ votes tended to have raised suicide rates. This highlights the need for policy, including suicide prevention strategies, to be specifically tailored to regional and local circumstances and needs.

Our findings are broadly consistent with those reported by Koltai et al from their recent study of ‘deaths of despair’,^15^ in which ecological correlations between level of support for Brexit and increases in rates of suicide and drug-related death were found at local authority population level. However, while Koltai et al examined changes between periods before and after the 2008–2009 recession, our cross-sectional design enabled us to focus on suicide rates for the 3 years that were temporally closest to the 2016 national referendum – i.e. the years immediately before, during and after it. In doing so we observed a potential influence of anomie on suicide rates that could be linked specifically with the Brexit phenomenon. Furthermore, our study examined the ecological association for the population of Greater London versus that of the rest of England separately. These two aspects of our investigation are novel.

The UK general election that was held on 12 December 2019 resulted in large increases in the number of seats won by the Conservative Party, alongside significant losses of Labour Party seats. Many of these Labour losses were in areas with strong ‘Leave’ voting preferences, suggesting that a sizeable proportion of traditional Labour voters who felt ‘left behind’ switched their support to the Conservative Party, likely because of its promise of a swift withdrawal from the EU following the protracted withdrawal deliberations. Increasing levels of support for populist policies may be driven partly by widening socioeconomic gaps in health.^26^ Poor health has been linked to a lack of control over life choices, likely to be intensified by shrinking welfare states and public services resulting from economic austerity.^26^ Subsequently, people may be more likely to reject conventional politics and vote for populist policies that promise radical and profound change.^26^

### Implications

Area-based factors are important potential targets for suicide prevention. A range of policy-level interventions may have an impact on suicide rates. Following the deep economic recession in 2008–2009, increases in suicide and self-harm rates were avoided in countries where generous unemployment welfare was in place.^27,28^ It is possible that welfare programmes have an impact on both unemployed people and those in work but fearing job loss.^27^ Financial anxiety, stigma and social isolation have been proposed as mechanisms by which unemployment affects mental health.^29^ It is likely that deprivation and social fragmentation are intertwined. Policies that address the factors associated with deprivation may help to shield vulnerable populations from being affected by major societal upheaval resulting from political change.

In the absence of more detailed information we can merely speculate as to why local authority populations in Greater London that returned a low ‘Leave’ vote in the 2016 referendum also tended to have a higher suicide rate and vice versa. The answer may lie with varying socioeconomic compositions and ethnic profiles of the local authority populations in Greater London, or with differing levels of contextual factors such as social cohesion/fragmentation.^9,19,20^ The sharply contrasting findings between Greater London and the rest of England that our study generated highlight the importance of tailoring policy according to specific regional contexts. Current guidance recommends that local areas produce their own bespoke suicide prevention strategies.^30^ Strategies should also consider the welfare and social needs of communities, perhaps paying particular attention to potential exacerbations in deprivation and social fragmentation in the aftermath of Brexit.

There is little published evidence to date regarding the potentially harmful psychological impact of the Brexit era, although one examination of the UK Household Longitudinal Study (‘Understanding Society’) reported a rise in average levels of psychological distress following the referendum, with no apparent difference in patterns of association observed between people who voted to ‘Leave’ versus those who voted to ‘Remain’.^31^ The study's authors concluded that levels of subjective well-being may have been both a cause and consequence of the slender national majority vote in favour of Brexit in 2016. These findings, as well as the disquieting correlations that our investigation has revealed, indicate a need for investigation of the psychological sequelae of the societal divisions and insecurities that have arisen before, during and following the 2016 national referendum.

Some published commentaries on the likely societal impact of various Brexit scenarios raise concerns about detrimental effects on population health, the National Health Service and the economy.^32–34^ Although the precise consequences of Brexit are unclear, they could include short-term increases in economic uncertainty,^35^ which could have a further impact on unemployment and job security. In this ecological study we were only able to examine correlates between Brexit-voting behaviour and suicide rates. Future studies could examine the consequences of Brexit on future rates of mental disorder and suicide, including area-based differences.

### Strengths and limitations

The key strength of our study lies in its utilisation of routinely reported national data-sets with equivalent measurements for each local authority population examined and with minimal levels of missing data. Thus, just a small fraction (1.9%) of all English local authorities was excluded entirely from the analyses. The excluded authorities had some of the smallest resident populations in the country, including the City of London and the Isles of Scilly, and so their omission from the study data-set will have had no material impact on the observed ecological correlations.

In conducting multivariable modelling we had to omit approximately a tenth (29/286, 10.1%) of all the local authority populations for the regions in England outside of Greater London because of small event counts in relation to the covariate that measured the ethnic profiles of these populations. However, as the results presented in Table 2 indicate, omission of this relatively small number of local authorities did not introduce a major selection bias for the multivariable modelling.

The most important limitation of this ecological, cross-sectional study was that we could not infer causal relationships from any of the correlations observed. Low education level has been identified as having a strong association with voting to leave the EU.^36^ We did not include educational attainment in our study because no suitable area-based measure was available for voting-age adults in the time period 2015–2017. However, the three factors in our model – area-level deprivation, percentage of the population aged 65 years and older, and percentage of the population that was White British – may have captured some of the covariance attributable to education level.

## Data Availability

The data underlying the results presented in the study are available from the UK's Electoral Commission website (https://www.electoralcommission.org.uk) and the online Public Health England Local Authority Health Profiles (https://fingertips.phe.org.uk/profile/public-health-outcomes-framework/data).
